# Staff Perspectives on Non‐Routine Compression Therapy for Inpatients With Venous Leg Ulcers: A Qualitative Study

**DOI:** 10.1111/iwj.70976

**Published:** 2026-06-15

**Authors:** Yaping Lian, Linda Birt, Fiona Poland, Felix Naughton, Christine Moffatt, David Wright

**Affiliations:** ^1^ University of Leicester Leicester UK; ^2^ Tissue Viability Team, Cambridge University Hospitals NHS Foundation Trust Cambridge UK; ^3^ School of Healthcare, College of Life Sciences, University of Leicester Leicester UK; ^4^ School of Health Sciences, University of East Anglia Norwich UK; ^5^ University of Nottingham Nottingham UK; ^6^ Nottingham University Hospital Nottingham UK; ^7^ School of Healthcare University of Leicester Leicester UK; ^8^ Health Services Research, School of Healthcare, College of Life Sciences University of Leicester Leicester UK

**Keywords:** compression bandage, compression therapy, hospital clinicians, qualitative, venous leg ulcer

## Abstract

Compression therapy is the evidence‐based treatment for healing venous leg ulcers. However, it is not routinely applied in many UK hospitals. This paper explores hospital staff's' perspectives of venous leg ulcer care provision where compression therapy is not routinely applied. A semi‐structured interview study was conducted with 11 participants, drawn from a larger study, who confirmed that their respective hospitals did not apply compression therapy to inpatients with venous leg ulcers. The interviews were analysed using reflexive thematic analysis. Findings indicate that venous leg ulcer care in hospitals was de‐prioritised, which, along with structural and organisational constraints, affected clinicians' engagement and focus on it in their hospitals. This de‐prioritisation also led to a lack of formal leg ulcer training in hospitals. However, some clinicians showed remarkable empathy for patients derived from their clinical professionalism and deep understanding of their suffering. To help hospital clinicians and senior managers prioritise venous leg ulcer care, it is crucial to first understand their clinical practise priorities. Secondly, understanding how some hospitals implement compression therapy could be beneficial for those where it is not routine practise. Further research should focus on better understanding clinicians and senior managers' clinical priorities and the structural and organisational constraints in real‐world settings, ideally in hospitals where compression therapy is a routine practise. Most importantly, the study highlighted key questions for hospital leaders and policymakers: consider fostering the established clinicians' empathy before it is too late.

## Key Points

- This study found that hospital clinicians face significant barriers to implement compression therapy for inpatients with venous leg ulcers due to organisational priorities and structural constraints.
- To address these barriers, we need to examine these organisational and structural factors that shape their practice priorities. Understanding how hospitals that routinely use compression therapy operate could provide insights into whether similar changes can be implemented in those that do not.
- The study also highlighted key questions for hospital leaders and policymakers: consider fostering clinicians’ empathy for patients with venous leg ulcers before it's too late.

## 1 Introduction

A leg ulcer is defined as an open wound between the knee and ankle joint that persists for at least 4 weeks [1]. Venous leg ulcers (VLUs) are the most prevalent type, accounting for over 70% of all leg ulcer cases [2]. Scientific evidence and national guidelines consistently recommend compression therapy as the fundamental, first‐line effective evidence‐based treatment for healing and managing VLUs [1, 3, 4, 5, 6, 7]. Compression therapy is a medical intervention that involves applying pressure to the lower limbs using bandages, stockings, hosiery or wraps [4]. The therapeutic efficacy of compression therapy in managing VLUs has been acknowledged for centuries, evolving from simple bandaging techniques to contemporary evidence‐based interventions for managing VLUs [8]. Compression therapy can alleviate pain, enhance healing rates, improve quality of life, mobility and functional status [1, 3, 4, 5, 6, 9]. Clinical evidence demonstrates that compression therapy can heal up to 83% of venous leg ulcers and prevent ulcer recurrence by 70% [10, 11, 12]. Despite strong evidence, patients with VLUs are frequently not treated with compression therapy in acute hospital settings [13]. A literature review conducted in 2020 indicated that compression therapy is widely applied in community settings, but is used less in secondary care [14]. When patients with VLUs are admitted to hospitals, their compression therapies are often discontinued [11, 13].

Inpatient venous leg ulcer management could differ from community care for several reasons. Firstly, inpatient management of venous leg ulcers usually happens in hospital wards where patients are admitted for acute conditions and often have complex medical needs. When hospitalised patients are admitted for acute conditions, it may overlook chronic venous leg ulcer care. Secondly, ward nurses often work in time‐pressured, task‐driven environments, while community nursing teams provide planned visits with more time for thorough assessment, regular review, and continuity of care in patients' homes, local health centres, or residential homes. Finally, community nurses often have greater expertise in leg ulcer management and follow structured pathways, resulting in more consistent and effective compression therapy in community settings than on hospital wards.

A systematic review conducted in 2023 identified only four studies exploring hospital clinicians' views and experiences of using compression therapy for inpatients with venous leg ulcers [15]. Three key themes were generated: educational needs arising from limited clinical procedure knowledge, organisational resources including the availability of suitable equipment and trained staff, and patient factors influencing adherence [15]. Although these four studies provided some insights, the study participants were situated in diverse community and hospital settings which may have hindered the reporting of perspectives from hospital clinicians. A subsequent national online survey of wound care specialists revealed that only 32% (32/101) of respondents applied compression therapy to their patients during hospital stays [13]. This significant disparity in the provision of sustained compression therapy for inpatients with VLUs is concerning [14]. To address this research gap, this study aims to examine hospital staff's perceptions of venous leg ulcer care provision where compression therapy is not routinely applied.

## 2 Methods

### 2.1 Study Design

A qualitative study involving hospital healthcare professionals and senior managers in secondary care was conducted over a 1‐year period from May 2024 to June 2025. The Consolidated criteria for reporting qualitative research (COREQ) checklist was used as a checklist for explicit and comprehensive reporting of this semi‐structured interview study (Appendix A) [16].

### 2.2 Methodological Orientation

Qualitative methodology is an appropriate research approach, which seeks and interprets how hospital staff contextualise their environment to understand why compression is little used in secondary care [17, 18, 19]. The study utilised semi‐structured interviews to delve into clinicians' views and experiences across different clinical roles in diverse hospital environments. This is because, to truly understand the reasons behind its limited use, it is crucial to engage with and explore clinicians' and senior managers' diverse views and experiences across various hospitals and clinical roles. Clinicians, as care providers, have the experience, knowledge and understanding of hospital care. Their experiences are particularly relevant within the context of hospital settings, cultures and practises, which can be explored through listening to their talks and asking questions. To strengthen the study design, input was sought from Patient and Public Involvement (PPI) members who were patients living with VLUs or caring for someone with them. Their insights during the study preparation phase significantly influenced recruitment, emphasising the importance of diverse participant representation across various hospital types of job roles and working experiences within NHS settings.

### 2.3 Eligibility Criteria

The inclusion criteria were hospital clinicians and senior managers who are involved in the care for inpatients with VLUs. Participants were excluded if they were not employed in hospital settings. The participants for this paper are those who confirmed that their respective hospitals did not apply compression therapy for inpatients with VLUs.

### 2.4 Participant Sampling and Recruitment

Purposive sampling was used to select hospital clinicians and senior managers who were involved in caring for inpatients with VLUs based on key characteristics such as job role and clinical experience. Study advertisements were distributed to professional social media platforms, conference networking events and professional networks across the United Kingdom (UK). Professional social media platforms include Tissue Viability Nurses UK Facebook and Lower Limb Clinicians 2gether Facebook. Conference networking events include European Wound Management Association (EWMA) conference, Society of Tissue Viability conference, The Royal Society of Medicine Venous Forum Annual Scientific meeting, Wounds UK Annual conference, The Vascular Societies' Annual Scientific meeting and Leg Ulcer Forum. Professional networks include Society of Tissue Viability, Society of Vascular Nurses, Vascular Surgery Clinical Trials Network, British Geriatrics Society. Study advertisements included a Participant Information Sheet (PIS), along with a link and a QR code to invite potential participants to express interest in interview participation. The expression of interest and availability survey was conducted via a Microsoft Forms survey. The survey form collects information such as name, email address, job role, region of practising in the UK, name of the hospital where they are practising.

Once wound care specialists were recruited, the network sampling, also known as snowball sampling [20], was employed to encourage these specialists to forward the PIS link and QR code to potential participants, including nurses, doctors, and senior managers in their respective hospitals. While this method helped expand the participant pool, it is important to acknowledge that network sampling may inadvertently exclude potential participants outside of these established networks, who could offer valuable insights and experiences. To address this, the researcher's professional networks were also used to recruit doctors, nurses, and senior managers who care for inpatients with VLUs in their hospitals.

### 2.5 Consent to Participate

After reading the PIS, any potential participants could email the researcher to express their interests in taking part in interviews. The researcher then emailed the potential participants to arrange an informal telephone conversation regarding the participation. This telephone call provided an opportunity not only for the potential participants to ask any questions but also for the researcher to emphasise the implications of the protocol and check the potential participants against the eligibility criteria. Once the participant was eligible for the study, the researcher then sent a link by email to the participant to complete an online consent form prior to participation in the study. The researcher highlighted that the participant was free to withdraw from the study at any time with no obligation to give the reason for withdrawal.

### 2.6 Data Collection

Data were collected through semi‐structured one‐to‐one interviews. Interviews were conducted online via Microsoft Teams meeting platforms. Only the primary researcher and the participants were present at interviews. The interviews were digitally recorded and transcribed verbatim using Microsoft Teams' built‐in transcription function. Audio recordings were also captured via Microsoft Word's ‘Dictate’ feature, with an encrypted audio recorder serving as a backup. This approach allowed the primary researcher to remain fully engaged in the conversation, facilitating richer dialogue and comprehensive data collection. Following each interview, the transcripts generated by Microsoft Teams built‐in transcription function were verified by the primary researcher against the recordings, meticulously proofread and corrected for errors. Any participant‐identifiable information was removed to maintain confidentiality.

The interview topic guide (Appendix B) was developed in consultation with the PPI group and the research team. It was piloted with one academic supervisor, two clinicians, and one university Master's student with no significant modifications. Written informed consent was obtained before each interview. Field notes were meticulously documented, and interviews were digitally recorded and transcribed verbatim. These transcripts were compared with the recordings to ensure accuracy, and notes were recorded to capture initial impressions and reflections.

### 2.7 Data Analysis

The primary researcher organised and managed the interview transcripts using Microsoft Word documents and data management software QSR Nvivo Version 14. Reflexive thematic analysis (TA) [21, 22, 23] was used to analyse the transcripts inductively. Reflexive TA is a method employed in qualitative research to identify, analyse, and report patterns or themes within data [22, 24, 25]. Reflexive TA is appropriate in this study as it enables me to recognise that my situated perspectives can help me notice, question and connect the meanings from the data [25]. This means the researcher's analysis orientation is an inductive process and my subjectivity is treated as a resource during the thematic analysis process [25]. This reflexive TA process involves six stages: familiarisation with the data, generation of initial codes, search for themes, review of themes, defining, and naming of themes [21].

Initially, the primary researcher familiarised herself with the data through conducting the interviews, reading the transcripts repeatedly and noting down initial thoughts. Then, the data were processed inductively into initial codes. These codes represented the core essence or meaning of sentences or paragraphs through symbolic words or short phrases from each transcript [26, 27]. This process involved immersing myself in the content and attentively observing emerging themes. This iterative engagement with the transcripts facilitated a more nuanced interpretation of participants' experiences and perspectives, thereby enhancing the rigour of the analysis. In accordance with the phases of reflexive TA [21], multiple cycles of sorting, defining and refining codes and themes were undertaken. During the mapping phase, the relationships and interactions between codes and themes were gradually established.

### 2.8 Data Saturation

Participant recruitment and analysis were conducted concurrently to facilitate constant comparison and identify similarities, differences, and patterns within the data. During data collection, new data was coded and categorised into themes, which were then compared to previously developed ones.

After analysing 23 interviews, the primary researcher decided to divide the dataset into two distinct groups: participants ‘applying compression therapy’ as a routine practise [28] and those ‘not applying compression therapy.’ This decision reflected the fundamentally different contexts of these two groups. This is because hospitals that apply compression therapy as a routine practise might operate within a framework of established clinical pathways and specialist staff training. In contrast, hospitals that do not apply it often face fundamentally different contexts, such as lack of specific policy guidance. For each dataset, emerging themes were regularly reviewed and discussed with the supervisory team to maintain analytical rigour and monitor for redundancy. Different geographic regions across all nations of the UK were also attempted.

For the dataset of participants ‘not applying compression therapy’, data saturation was reached at 11 interviews, when no new codes or themes were identified and findings consistently reinforced established patterns [29, 30]. Data saturation, also known as information redundancy, is a widely accepted concept in thematic analysis research [29]. This approach ensured a rich and comprehensive understanding of participants' perspectives, thereby enhancing the trustworthiness and depth of the findings [29].

### 2.9 Management of Research Data

Only the primary researcher had access to the full dataset generated by the study. The primary researcher's supervisory team had access to the pseudonymous dataset. All research team members complied with General Data Protection Regulation (GDPR) regarding data collection, storage, processing and disclosure of personal information. Data collected during the study included identifiable data (i.e., name, email address, telephone/mobile phone number) to enable the researcher to communicate with participants throughout the study. The primary researcher also collected demographic data (i.e., hospitals they are practicing) to ensure the sample is varied across the study. Only the primary researcher had the identifiable information about the participants. All information collected in the study were kept strictly confidential.

The University of xxx systems and networks were used for all data collection and storage. The primary researcher stored data on an encrypted secure drive accessible only to the research team. Electronic files containing identifiable information were filed using pseudonyms. The primary researcher stored personally identifiable information securely and separately from the research data as early as possible. The participants' information was marked with an ID number and not the name. The key for linking the ID number to the participants' identity was accessible only to the primary researcher.

Access to Microsoft Teams recordings could only be accessed via password by the primary researcher. The researcher anonymised all transcripts using unique participant reference numbers. Audio files were removed from the digital recording device upon encryption and filed securely. All audio/video recordings were deleted completely after transcription. Transcription and analysis were undertaken on a password‐protected computer. When reporting the results, any quotes were pseudonymous. Data were used solely for the purposes of this study.

### 2.10 Confidentiality

The primary researcher collected participants' characteristics such as name, job roles and duration of hospital experiences to enhance study transferability. To ensure the participants' anonymity and confidentiality was protected, the researcher removed the names of all participants and replaced them with pseudonyms in the electronic database. All personal identifiable information was removed in the final transcript. Some pseudonymous transcripts were shared with the research supervisory team during the data analysis process [31]. The researcher informed participants that she might use software to transcribe the data and then check the transcripts for accuracy. No participants were identified in publications to avoid breaking their confidentiality [32].

### 2.11 Quality and Rigour

The trustworthiness of this study was rigorously evaluated. Techniques such as obtaining samples from multiple UK hospitals, holding regular debriefing meetings with supervisors and receiving peer‐to‐peer scrutiny during analysis strengthened credibility, transferability and confirmability [33]. All supervisors (DW, FP, LB, FN) and academic peers (FF, CS, JM) challenged and verified interpretations of codes, subthemes and themes. Synthesised Member Checking (SMC) [34] further validated and assessed the trustworthiness of the qualitative results [35].

Furthermore, all stages of data were meticulously documented in Word and NVivo software, including coding decisions and theme development. This transparent record supported the dependability of findings. Beyond dominant themes, attention was paid to deviant cases challenging emerging interpretations. Rather than treating these as anomalies, these were incorporated as valuable insights into practise barriers and tensions. This approach enhanced credibility by demonstrating the analysis's account of data complexity and diversity, rather than seeking neat consensus. PPI members also contributed to discussions about codes and themes, assessing their resonance with their experiences. Preliminary key themes were presented at national and international conferences and shared with the study's Clinical and Academic Advisory panel.

### 2.12 Researcher and Reflexivity

The primary researcher conducted interviews and performed data analysis. The primary researcher was a female Tissue Viability Nurse Specialist (TVN) with 14 years of experience in acute hospital care. She conducted this study as part of her doctoral research. Her positionality as a clinician‐researcher was shaped by her extensive background in Tissue Viability, long‐standing experience within NHS hospital settings, and her identity as a female nurse specialist from a racially minoritised background. Her clinical background provided an insider's perspective on the hospital environment, enabling her to relate her clinical experiences to staff participants' experiences, language and challenges. This facilitated rapport and enabled her to interpret nuanced references to clinical practise and systemic constraints. However, this insider knowledge also carried the risk of reinforcing assumptions and prioritising perspectives that aligned with her professional wound care experience.

The primary researcher's relationships with interviewees were characterised by the use of professional language due to their shared field of clinical practise. The primary researcher knew some participants prior to the study, which helped foster trust and candid discussion. With participants the researcher had not met before, building rapport required intentional effort to create a space where they felt comfortable expressing honest and potentially critical views. These differing levels of familiarity produced varied interview dynamics, but all were grounded in a shared professional context that facilitated meaningful conversations about barriers and enablers to compression therapy in hospitals. However, the primary researcher's clinical experience working with patients with VLUs could potentially introduce unconscious bias when conducting interviews and analysing data. To address this, the initial interview transcripts were reviewed by one academic supervisor (LB) to identify any leading questions. The supervisory meetings facilitated the identification of areas for improvement and other opportunities for subsequent interviews. The primary researcher maintained a reflexive journal documenting her reflections after each interview and her thought process while reading transcripts, generating initial codes, themes and identifying areas to refine her interviewing techniques.

## 3 Results

### 3.1 Participants Characteristics

A total of 38 hospital staff expressed interest in the study, in which two participants withdrew from the study and six did not participate due to clinical time pressures. Out of the 30 participants, 11 participants stated that compression therapy was not a routine clinical practise in their respective hospitals, which is the sample for this paper.

These interviews lasted on average for 1 h and 50 s and were audio & video recorded and transcribed. Out of 11 participants, 9 (82%) were female. Participants with over 20 years of work experience comprised the majority (7, 64%), followed by equal participants with 5–9 and 10–19 years of experience (2, 18% per grouping). There were an equal number of Vascular Nurses, Tissue Viability Nurses, Senior Managers and other specialist nurses (2, 18% per grouping). There were three (27%) other clinicians including a Healthcare Assistant, Lymphoedema Consultant, and Vascular Surgeon (1, 9% per grouping). Each profession contributed particular perspectives, reflecting their distinctive roles in patient care. There were an equal number of participants from the North, South of England, London (1, 9% per grouping), with the East of England having the most participants (7, 64%). There was only one participant from Wales. There were three participants working in general hospitals and eight in teaching hospitals (Table 1). To ensure confidentiality, participants' identifying information were removed.

**TABLE 1:** Participant demographics and clinical characteristics (*n* = 11).

Variable	*n*	%
Participant profession		
Vascular nurses	2	18%
Vascular surgeon	1	10%
Tissue viability nurse	2	18%
Senior manager	2	18%
Other specialist nurses	2	18%
Other clinicians	2	18%
Hospital working experience		
5–9 years	2	18%
10–19 years	2	18%
More than 20 years	7	64%
Geographic location		
East of England	7	64%
London	1	9%
North of England	1	9%
South of England	1	9%
Wales	1	9%
Hospital type		
Teaching hospital	8	72%
General hospital	3	27%

### 3.2 Main Findings

In the dataset of hospital staff where compression therapy was not routinely applied, three key themes were generated from the data:

1. De‐prioritisation of Venous Leg Ulcer Care.
2. Structural and Organisational Constraints.
3. Empathy for Patient Care.

#### 3.2.1 Theme 1: De‐Prioritisation of Venous Leg Ulcer Care

Participants' accounts indicated that VLU care provision in hospitals appeared to be de‐prioritised by institutional prioritisation of pressure ulcers, which was positioned as a dominant clinical and organisational focus within the trust and the Tissue Viability team, as stated by the senior manager:

Hospital acquired pressure ulcers is … massive priority for the trust … [and] for the [Tissue Viability] team. (P16, Senior Manager, Teaching Hospital)

This was echoed by the TVN (P02) who expressed that ‘They [patients with VLUs] are just being brushed off really [in hospitals], you don't need that [compression treatment].’ This TVN further explained that this prioritisation of pressure ulcer care was not accidental but financially incentivised through Commissioning for Quality and Innovation (CQUIN) targets: ‘There's … money influence with pressure ulcers [care], but there isn't with leg ulcers…’ (P02, TVN, Teaching Hospital). The TVN (P02)'s account could indicate that the CQUIN targets make pressure ulcer prevention a funded and highly visible performance metric, while venous leg ulcer care remained financially invisible.

Consequently, the de‐prioritisation of VLU care in this context is seen as an active organisational process where specific service is systematically sidelined in favour of competing institutional targets. Rather than a passive lack of attention, it represents a structural tension where resource constraints and external performance metrics necessitate the downgrading of venous leg ulcer care delivery in hospital settings.

Some participants stressed that ward nurses' VLU care knowledge and skill could be contributed to by the de‐prioritisation of VLU care in hospitals. They reported ‘some *very questionable bandaging techniques … a bit shocking*’ (P03, Vascular Practitioner, Teaching Hospital), suggesting inconsistencies in leg ulcer clinical standards. The ward nurses' questionable bandaging techniques may be attributed to a lack of formal training as one TVN stated ‘They [ward nurses] get very, very little [formal training] on wound care from the Tissue Viability team … small pockets of teaching here and there’ (P02, TVN, Teaching Hospital). The difficulty in securing approval for leg ulcer training for ward nurses was also highlighted by a senior manager: ‘Getting that [leg ulcer training] approved [forward nurses] … is quite difficult’ (P20, Senior Manager, Teaching Hospital).

The lack of formal training for TVNs was also identified by participants. TVN (P04) explained that their leg ulcer knowledge primarily stemmed from industry‐led E‐learning as ‘*there was no training provided by the trust…*’ (P04, TVN, Teaching hospital). As a result, participants revealed that compression therapy, which is the core element of venous leg ulcer management, is not within hospital TVNs' routine clinical repertoire:

They [TVNs] are not qualified to apply compression … because they've not had any formal leg ulcer training … that's the issue with the TVNs in our trust. They are not…leg ulcer trained. (P03, Vascular Practitioner, Teaching hospital)

The ‘de‐prioritisation of VLU care’ was also reflected in the practitioner's level, where the low prestige and invisibility of VLU care was highlighted by vascular surgeon (P13):

It's very sexy and exciting to put in … stent for someone with aneurysm … whereas treating … [venous] leg ulcers and getting them healed… is not as important. (P13, Vascular Surgeon, Teaching Hospital)

The vascular surgeon here contrasted the prestige of high‐tech, high‐cost interventions such as aneurysm repair with the perceived mundanity of healing leg ulcers in vascular field.

#### 3.2.2 Theme 2: Structural and Organisational Constraints

In healthcare, the structural and organisational constraints refer to the features of the system that limit what clinicians can do. These constraints shape behaviour and decision‐making. Participants highlighted structural and organisational constraints in hospital settings, where limited staffing capacity and increasing workloads intersected to constrain the consistent provision of evidence‐based care.

#### 3.2.3 Limited Staffing Capacity

Participants identified limited staffing capacity across both specialist and ward‐based nurses' workforce, which could contribute to the difficulty in improving leg ulcer care in hospitals. They stated that TVNs were the logical leads for advancing best practise in VLU care; however, a TVN explained that their capacity was ‘*too small*’ to provide the necessary training, support, and direct patient care for inpatients with VLUs:

Tissue viability would be a logical start, but … their numbers are so small, there need to be … funding … to have … more nurses [TVNs] to be able to offer that [compression therapy] …. (P02, TVN, Teaching Hospital)

Some participants described ward nurses, who might otherwise provide continuity of care, were also overstretched, with ‘*not enough*’ staff to deliver consistent leg ulcer care (P12, Other specialist nurse, General Hospital). In addition, limited staffing capacity and high staff turnover in hospitals could also have an impact on the development of skills in leg ulcer care:

Over the years we've done a lot of training for ward staff … a lot of engagement and people are keen to do it … they get trained up … get competent … they move on to different job roles … you're back to square one with no one on the ward competent to do it …. (P12, Other specialist nurse, General Hospital)

Thus, workforce fragility was seen as a key structural barrier to embedding compression therapy into routine hospital practise.

#### 3.2.4 Increasing Workloads

Participants stressed that limited staffing inevitably cascaded into increasing workloads for both ward and specialist nurses. Some participants described the hospital system under constant strain, with ‘*They [ward nurses] are expected to do …a lot more with the same or less staff as they did 10‐15 years ago*…’ (P17, Other specialist nurse, Teaching Hospital). The increasing workloads were not confined to ward staff but extended to specialist clinicians, who described ‘*It*'*s quite a big workload. I see all in and outpatients … I have outpatient clinics as well for [inpatient] complex patients*’ (P12, Other specialist nurse, General Hospital). As a result, this specialist nurse was not able to provide compression therapy for inpatients with VLUs.

Furthermore, communication barriers between hospital and community systems exacerbated workload pressures. Fragmented IT infrastructures meant hospital staff had to duplicate documentation and chase information manually, creating inefficiencies and frustrations. Consequently, hospital staff are ‘*wasting time for calling GP surgeries*’ (P17, Other specialist nurse, Teaching Hospital), detracting from already stretched clinical time.

#### 3.2.5 Theme 3: Empathy for Patients

Across the dataset, participants expressed a deep sense of empathy for patients with VLUs. This empathy was rooted not only in clinical professionalism but a deep understanding of patients' suffering as their lives were deeply shaped by chronic leg ulcers. They articulated a strong awareness of the long‐term burden VLUs place on patients. They emphasised that without compression therapy, many patients were caught in cycles of recurrence, pain and dependency, as stated by a vascular nurse (P09):

Vascular patients get a bit of an ordeal… They have very chronic ulceration … not easy to deal with … end up becoming patients for life… that's what I feel passionate about is, making life better for them …. (P09, Vascular Nurse Specialist, General Hospital)

By framing patients' experiences as an ‘*ordeal*’, participants positioned their professional empathy as a counterbalance to the systemic neglect and organisational de‐prioritisation of VLU care. In addition, participants also recognised serious consequences for patient safety and quality of life, emphasising the significant disruption to patients' lives, routines and independence:

Life is difficult for them. It's challenging if they've got wounds [venous leg ulcers] … mucky, smelly. It impacts everything on the quality of life…. (P09, Vascular Nurse Specialist, General Hospital)

Some participants view the absence of compression care provision as ‘*poor care*’ and ‘*shameful*,’ which could lead to wound deterioration, infection, immobility and repeated readmissions (P02, TVN, Teaching Hospital). For other participants, their empathy for patients was framed through the language of resilience. The vascular surgeon described the endeavour as a *‘hard fight’* (P13, Vascular Surgeon, Teaching Hospital), yet one worth pursuing. This narrative of determination suggests that clinicians saw themselves not only as care providers but also as advocates resisting the organisational and cultural forces that limit the visibility and priority of VLU care.

Another strand of empathy was expressed through participants' emphasis on teamwork and collaboration. *‘If there's a place for us to help, then we will, because we're all working towards patients' care…*’ (P17, Other specialist nurse, Teaching Hospital). Empathy here was not an individualised endeavour, but a collective commitment to patient‐centred care. This collaborative ethos reflected a willingness among clinicians to transcend professional boundaries, recognising that effective care for VLUs requires shared responsibility across disciplines and departments.

## 4 Discussion

This study explored the perceptions of hospital staff regarding VLU care provision in UK hospitals where compression therapy was not a routine practise. The findings highlighted that the de‐prioritisation of VLU care, alongside the structural and organisational constraints, could have influenced clinicians' engagement and clinical focus on VLU care within their hospitals. Nevertheless, clinicians exhibited profound empathy for patients, derived from their clinical professionalism and deep understanding of patients' suffering.

Our study revealed a significant disparity in wound care practises in hospital settings, where pressure ulcer care takes precedence over VLU care. This finding was echoed in our sister study [28]. Over the last 15 years, there has been a national drive to reduce hospital‐acquired pressure ulcers, leading to increased surveillance and reporting by TVNs [36]. This shift is supported by national policies and quality improvement initiatives that prioritise pressure ulcer reduction as a key patient safety indicator [37].

While both pressure ulcers and VLUs represent significant patient safety concerns, they exist within different frameworks of institutional accountability. Pressure ulcers are subject to rigorous NHS reporting mandates and potential financial penalties as hospital‐acquired harms [37]. Furthermore, there is an inherent institutional recognition that pressure ulcers can result from negligent practises, making them a primary driver for litigation and legal liability [38]. Consequently, the prioritisation of pressure ulcer prevention often functions as a defensive risk management strategy. As noted by our participants, the organisational ‘de‐prioritisation’ of VLU care is a byproduct of this asymmetrical accountability. While the external performance and legal pressures for pressure ulcers create a structural focus in hospitals, this is currently absent for chronic VLU outcomes, leading to an inevitable disparity in resource allocation and management focus.

Subsequently, when VLU care is de‐prioritised in hospital settings despite its detrimental impact on patients' health and quality of life, the omission of compression therapy for patients with VLUs will not be afforded the same level of concern as pressure ulcer care, which is widely recognised [28]. This de‐prioritisation could contribute to the lack of formal training opportunities for hospital TVNs and ward‐based nurses. Our study found that if TVNs wanted to enhance their skills in VLU care they relied on ad‐hoc commercial training, which could risk variability in clinical standards and practise, missing opportunities to equip TVNs with the depth of knowledge needed to lead on compression therapy and VLU care in hospitals.

Structural and organisational constraints such as limited staffing capacity and high workloads can reduce workforce and create inefficiencies in care delivery [39]. Understaffing has been reported as a key factor adversely impacting hospital performance, patient experience, and staff satisfaction [39, 40, 41, 42, 43]. Gayle et al. [41] also provided evidence of lower staffing levels being associated with higher adverse outcomes, which could contribute to operational uncertainty, increased pressure on remaining staff, and potential inequities in patient care [44]. Our study found that nurses had to manage increasing patient loads without corresponding staffing, creating a chronic overload environment that compromises essential non‐urgent care like compression therapy. The Royal College of Nursing suggests that excessive workload could exacerbate nurse recruitment and retention, leading to a workforce sustainability crisis [42]. The ongoing cycle of loss and replacement of nurses undermines the development of a stable, skilled workforce, which is crucial for maintaining the skillsets for compression therapy delivery in hospitals [42]. As a result, the challenging staffing capacity and greater workloads could erode the emotional and professional fulfilment traditionally associated with nursing, leading to job dissatisfaction, emotional exhaustion, further high turnover, and loss of experienced staff [39]. Furthermore, frequent staff turnover could also hinder nursing skill competencies in routine ward practise, undermining capacity‐building and training efficacy [43]. A review showed that nurse staffing levels have a direct impact on patient outcomes in hospitals [42].

Our findings indicate that clinicians in hospitals have profound empathy for patients with VLUs. They understand the impact of VLUs on patients' quality of life. They recognise that the impact was not only clinically severe symptoms from unmanaged or poorly managed ulcers, but also that stigma, embarrassment, and social withdrawal were associated with their poor management [45, 46]. The concept of empathy is a common denominator for healthcare professionals such as nurses and doctors [47]. It is often related to emotion, conscientiousness, care, communication and support [48]. Empathy is also regarded as the ability to understand another person's experience, feelings and thought [47]. Empathetic professionals comprehend the needs of patients who feel safe to express their thoughts and problems that concern them [47].

However, literature reported that certain factors could negatively influence clinicians' development and fostering of empathy [47]. These include a high volume of patients that professionals must manage, the lack of adequate time, and focus on therapy within the existing academic culture [47]. This could mean that the de‐prioritisation of VLU care in hospital settings and structural and organisational constraints could adversely impact clinicians' empathy towards patients' VLU treatment in hospitals. Given that healthcare professionals with high levels of empathy could operate more efficiently to fulfil their role [47], the key question for hospital leaders and policymakers is how to foster the established clinicians' empathy before it is too late.

### 4.1 Study Strengths and Limitations

This study offers novel insights into the challenges of applying compression therapy for inpatients with VLUs in acute hospital settings. A key strength lies in the inclusion of a variety of hospital‐based clinicians and senior managers across different specialties and roles, which helped provide a range of perspectives. This breadth of viewpoints allowed for a comprehensive understanding of organisational and professional influences shaping VLU care. Nevertheless, some limitations should be acknowledged. The findings are based on participants' accounts of their experiences and do not necessarily reflect their actual practises or decision‐making in clinical settings. In addition, social desirability bias [49, 50] may have influenced participants' accounts, given the sensitivity of discussing care omissions and organisational pressures. While snowball sampling allowed access to busy hospital clinicians caring for patients with VLUs, we acknowledge the potential for the lack of diversity [51, 52]. To mitigate this, we purposefully recruited participants from different geographic regions across the UK to ensure a wider range of institutional perspectives.

Finally, while the sample size of 11 participants is relatively small, however, in qualitative inquiry, the focus is on data saturation when no new codes or themes were identified and findings consistently reinforced established patterns [29, 30], rather than statistical representativeness. This cohort of participants represents a purposive subset drawn from a larger study, specifically selecting for a heterogeneity of role, including vascular surgeons and senior managers, to capture a multi‐dimensional view of organisational barriers. While the professional diversity strengthens the internal validity of the themes, the geographic focus on discrete areas of England might limit the transferability of the study findings across the UK. Nevertheless, this sample size is consistent with empirical evidence from Guest et al. [53], who demonstrated that thematic saturation typically occurs within the first 12 interviews, with basic elements for overarching metathemes present as early as six interviews. In this study, given the high degree of specialised expertise among the participants, data saturation was achieved as the subsequent interview in the sequence yielded no new codes or themes [53, 54]. These findings are further corroborated by our sister study [28], which identified echoing organisation constraints, suggesting a high degree of consistency and dependability in these findings.

### 4.2 Implications of the Findings

This qualitative study was the first to explore the accounts of hospital staff experiences on compression therapy in acute hospitals where compression was not routinely applied. Findings from our study indicate that the de‐prioritisation of VLU care coupled with organisational constraints could be the main barriers for hospital clinicians to implement compression therapy in hospital settings. To assist hospital clinicians and senior managers in prioritising venous leg ulcer care, it is important to first examine the organisational factors that shape their clinical practise priorities. Secondly, understanding the care structures and organisational priorities in hospitals where compression therapy is routinely used for patients could provide insight into whether similar changes could be made in hospitals where it is not used to support its use for inpatients with VLUs.

## 5 Conclusions

The study highlighted the de‐prioritisation of venous leg ulcer care alongside the structural and organisational constraints that could have influenced clinicians' engagement and clinical focus on VLU care within their hospitals. The de‐prioritisation of VLU care could also contribute to the lack of formal leg ulcer training in hospital settings. Nevertheless, some clinicians exhibited remarkable empathy for patients, derived from their clinical professionalism and deep understanding of patients' suffering. To help hospital clinicians and senior managers prioritise venous leg ulcer care, it is important to first examine their clinical practise priorities. Secondly, understanding how some hospitals implement compression therapy could also be beneficial for those where it is not a routine practise. Therefore, further research is to focus on better understanding clinicians and senior managers' clinical priorities and the structural and organisational constraints in real‐world settings. This could be conducted in hospitals where compression therapy is a routine clinical practise. Most importantly, the study highlighted key questions for hospital leaders and policymakers: consider fostering the established clinicians' empathy before it is too late.

## Funding

Yaping Lian was funded by a National Institute for Health and Care Research (NIHR) (NIHR302890) Doctoral Clinical and Practitioner Academic Fellowship (DCAF) (302890). The views expressed are those of the authors and not necessarily those of the NIHR or the Department of Health and Social Care.

## Ethics Statement

Ethical approval was granted by the University of Leicester (UoL) Medical and Biological Sciences Research Ethics Committee (Project ID: 0257) and Health Research Authority (REC reference: 24/HRA/0574).

## Consent

Written consent was gained before each interview.

## Conflicts of Interest

The authors declare no conflicts of interest.

## Data Availability

The ethical approval for this study does not allow the raw data (interview transcripts) to be shared outside of the research team as it could potentially result in study participants being identified.

## Appendix A See Table A1

**TABLE A1:** Consolidated criteria for reporting qualitative studies (COREQ): 32‐item checklist.

Item no	Guide questions/Description	Reported on Page #
Domain 1: Research team and reflexivity		
Personal Characteristics		
1. Interviewer	Which author/s conducted the interview?	pg. 11
2. Credentials	What were the researcher's credentials?	pg. 11
3. Occupation	What was their occupation at the time of the study?	Pg 11
4. Gender	Was the researcher male or female?	Pg 11
5. Experience and training	What experience or training did the researcher have?	Pg 11
Relationship with participants		
6. Relationship established	Was a relationship established prior to study commencement?	Pg 11
7. Participant knowledge of the interviewer	What did the participants know about the researcher? e.g., personal goals, reasons for doing the research?	Pg 11
8. Interviewer characteristics	What characteristics were reported about the interviewer/facilitator? e.g., Bias, assumptions, reasons and interests in the research topic	Pg 11–12
Domain 2: study design		
Theoretical framework		
9. Methodological orientation and Theory	What methodological orientation was stated to underpin the study? e.g., grounded theory, discourse analysis, ethnography, phenomenology, content analysis	Pg 4–5
Participant selection		
10. Sampling	How were participants selected? e.g., purposive, convenience, consecutive, snowball	Pg 5–6
11. Method of approach	How were participants approached? e.g., face‐to‐face, telephone, mail, email	Pg 6
12. Sample size	How many participants were in the study?	Pg 12–13
13. Non‐participation Setting	How many people refused to participate or dropped out? Reasons?	Pg 12
14. Setting of data collection	Where was the data collected? e.g., home, clinic, workplace	Pg 6–7
15. Presence of nonparticipants	Was anyone else present besides the participants and researchers?	Pg 6
16. Description of sample	What are the important characteristics of the sample? e.g., demographic data, date	Pg 12–13
Data collection		No
17. Interview guide	Were questions, prompts, and guides provided by the authors? Was it pilot tested?	Pg 7
18. Repeat interviews	Were repeat interviews carried out? If yes, how many?	N/A
19. Audio/visual recording	Did the research use audio or visual recording to collect the data?	Pg 6–7
20. Field notes	Were field notes made during and/or after the interview or focus group?	Pg 6–7
21. Duration	What was the duration of the interviews?	Pg 12
22. Data saturation	Was data saturation discussed?	Pg 8–9
23. Transcripts returned	Were transcripts returned to participants for comment and/or correction?	Pg 10
Domain 3: analysis and findings		
Data analysis		
24. Number of data coders	How many data coders coded the data?	Pg 7–8
25. Description of the coding tree	Did the authors provide a description of the coding tree?	N/A
26. Derivation of themes	Were themes identified in advance or derived from the data?	Pg 14
27. Software	What software, if applicable, was used to manage the data?	Pg 7
28. Participant checking	Did participants provide feedback on the findings?	Pg 10–11
Reporting		
29. Quotations presented	Were participant quotations presented to illustrate the themes/findings? Was each quotation identified? E.g., participant number	Pg 14–18
30. Data and findings consistent	Was there consistency between the data presented and the findings?	Pg 14–18
31. Clarity of major themes	Were major themes clearly presented in the findings?	Pg 14–18
32. Clarity of minor themes	Is there a description of diverse cases or a discussion of minor themes?	Pg 14–18

*Source:* See Tong et al. [16].

## Appendix B Interview Topic Guide

### OCTOPUS Study

Optimising Compression Therapy for inpatients with venous ulcers.

### Interview Topic Guide

Welcome and introduction.

Thank you for agreeing to take part in the interview. I appreciate you are very busy, so the interview will not take longer than 1 h.

I would like to highlight the confidentiality of the interview that everything you tell me will be kept confidential unless you tell me something that indicates danger to yourself or others or poor practises, in which case, it would need to be reported, but I will discuss that with you.

- The recording will be deleted after being transcribed
- You won't be identified individually in any report.
- All information will be anonymised
- We will not tell anyone else, including your employer organisation, what you tell us as an individual.
- All your views are of value to us. There are no right or wrong answers
- Please ask me to clarify if the question isn't clear.
- We remind you not to share any personal or patient identifying information during this interview
- Occasionally, I might look down to take notes; please assured that I am still actively listening.

**Table:**

What are your experiences of managing leg ulcers in your hospital?	
What treatment did you use in your hospital?	
What kind of policy and guidelines do you have for the care of patients with leg ulcers in your hospital?	
How did you learn about the skills and knowledge for patients with venous leg ulcers?	
What are your experiences of using compression therapy in hospitals?	
Some experience that it can be difficult to implement compression therapy in hospitals.	
What do you think might help address these views?	
How confident do you feel about the use of compression in your hospital?	
What other areas do you think might have an impact on the use of compression therapy in your hospital?	

Anything not covered? Is there anything that we haven't covered in the interview that you think we should know or think about?

Closing and thanks—I know a lot of what we talk about is kind of sensitive, so I would just like to check that you are ok and that you are still happy for me to use all the information provided. If you like, you could ask to erase any sections of the recording.

Thank them for their time and contribution.
